# Efficacy, safety and population pharmacokinetics of sapropterin in PKU patients <4 years: results from the SPARK open-label, multicentre, randomized phase IIIb trial

**DOI:** 10.1186/s13023-017-0600-x

**Published:** 2017-03-09

**Authors:** Ania C. Muntau, Alberto Burlina, François Eyskens, Peter Freisinger, Corinne De Laet, Vincenzo Leuzzi, Frank Rutsch, H. Serap Sivri, Suresh Vijay, Milva Orquidea Bal, Gwendolyn Gramer, Renata Pazdírková, Maureen Cleary, Amelie S. Lotz-Havla, Alain Munafo, Diane R. Mould, Flavie Moreau-Stucker, Daniela Rogoff

**Affiliations:** 1 University Children’s Hospital, University Medical Center Hamburg Eppendorf, Martinistrasse 52, D–20246 Hamburg, Germany; 2 University Hospital, Padova, Italy; 3 Universitair Ziekenhuis Antwerpen, Antwerp, Belgium; 4 Children’s Hospital Kreiskliniken, Reutlingen, Germany; 5 Hôpital Universitaire des Enfants Reine Fabiola, Brussels, Belgium; 6 Universita La Sapienza, Rome, Italy; 7 Muenster University Children’s Hospital, Muenster, Germany; 8 Hacettepe University School of Medicine, Ankara, Turkey; 9 Birmingham Children’s Hospital, Birmingham, UK; 10 Department of Pediatrics, University of Bologna, Bologna, Italy; 11 Centre for Paediatric and Adolescent Medicine, Division for Neuropaediatrics and Metabolic Medicine, University of Heidelberg, Heidelberg, Germany; 12 University Children’s Hospital, Prague, Czech Republic; 13 Great Ormond Street Hospital, London, UK; 14 Dr. von Hauner Children’s Hospital, Munich, Germany; 15 Merck Institute for Pharmacometrics, Lausanne, Switzerland; 16 Projections Research Inc., Phoenixville, USA; 17 EMD Serono, Billerica, MA USA

**Keywords:** Sapropterin, Phenylalanine hydroxylase, Phenylketonuria, Hyperphenylalaninemia, Pharmacokinetics, SPARK

## Abstract

**Background:**

Sapropterin dihydrochloride, a synthetic formulation of BH_4_, the cofactor for phenylalanine hydroxylase (PAH, EC 1.14.16.1), was initially approved in Europe only for patients ≥4 years with BH_4_-responsive phenylketonuria. The aim of the SPARK (Safety Paediatric efficAcy phaRmacokinetic with Kuvan®) trial was to assess the efficacy (improvement in daily phenylalanine tolerance, neuromotor development and growth parameters), safety and pharmacokinetics of sapropterin dihydrochloride in children <4 years.

**Results:**

In total, 109 male or female children <4 years with confirmed BH_4_-responsive phenylketonuria or mild hyperphenylalaninemia and good adherence to dietary treatment were screened. 56 patients were randomly assigned (1:1) to 10 mg/kg/day oral sapropterin plus a phenylalanine-restricted diet or to only a phenylalanine-restricted diet for 26 weeks (27 to the sapropterin and diet group and 29 to the diet-only group; intention-to-treat population). Of these, 52 patients with ≥1 pharmacokinetic sample were included in the pharmacokinetic analysis, and 54 patients were included in the safety analysis. At week 26 in the sapropterin plus diet group, mean phenylalanine tolerance was 30.5 (95% confidence interval 18.7–42.3) mg/kg/day higher than in the diet-only group (*p* < 0.001). The safety profile of sapropterin, measured monthly, was acceptable and consistent with that seen in studies of older children. Using non-linear mixed effect modelling, a one-compartment model with flip-flop pharmacokinetic behaviour, in which the effect of weight was substantial, best described the pharmacokinetic profile. Patients in both groups had normal neuromotor development and stable growth parameters.

**Conclusions:**

The addition of sapropterin to a phenylalanine-restricted diet was well tolerated and led to a significant improvement in phenylalanine tolerance in children <4 years with BH_4_-responsive phenylketonuria or mild hyperphenylalaninemia. The pharmacokinetic model favours once per day dosing with adjustment for weight. Based on the SPARK trial results, sapropterin has received EU approval to treat patients <4 years with BH_4_-responsive phenylketonuria.

**Trial registration:**

ClinicalTrials.gov, NCT01376908. Registered June 17, 2011.

**Electronic supplementary material:**

The online version of this article (doi:10.1186/s13023-017-0600-x) contains supplementary material, which is available to authorized users.

## Background

Hyperphenylalaninemia (HPA) is a rare inherited metabolic disorder caused by reduced activity of the hepatic enzyme phenylalanine hydroxylase (PAH, EC 1.14.16.1), which catalyses the conversion of phenylalanine (Phe) to tyrosine. Most cases of HPA (98%) in North American and European populations are due to mutations in the *PAH* gene but, in rare cases of HPA (1–2%), the cause can be a defect in the metabolism of the natural PAH cofactor, the R diastereoisomer of tetrahydrobiopterin (BH_4_) [1–3]. Owing to the reduced activity of PAH due to either mechanism, patients with HPA have an accumulation of Phe in the blood and body tissues and a relative deficiency of tyrosine and subsequent metabolites such as epinephrine [4, 5].

HPA can present with a spectrum of phenotypes that can be grouped into three main categories according to blood Phe concentrations before therapeutic intervention: classical PKU (Phe >1200 μmol/L); mild PKU (Phe 600–1200 μmol/L); mild HPA (Phe 120–600 μmol/L) [2, 5, 6]. PKU can lead to cognitive impairment and, if untreated, patients can develop mild-to-severe intellectual disability and other neurological sequelae [2, 5, 7].

The therapeutic range of Phe concentration varies according to different guidelines [8, 9], and there is no international consensus. The US diagnostic and management guidelines recommend that the initiation of treatment for PKU should be undertaken as early as possible, preferably within the first week after birth, with a goal of having blood Phe in the range 120–360 μmol/L within the first 2 weeks of life, to prevent permanent neurological damage [10]. The European guidelines recommend target concentrations of 120-360 μmol/L for individuals aged 0-12 years and for maternal PKU [11]. In both, this is largely achieved by a natural protein-restricted diet and Phe-free synthetic amino-acid supplementation [10, 11]. However, adherence to a Phe-restricted diet is burdensome owing to the need for long-term dietary counselling and daily micronutrient supplementation [12]. The management guidelines also stipulate that a course of treatment with BH_4_ should be investigated [10, 11].

Sapropterin dihydrochloride (sapropterin, Kuvan®, Merck, Geneva, Switzerland, an affiliate of Merck KGaA, Darmstadt, Germany, and BioMarin, Novato, CA, USA) is a synthetic formulation of BH_4_ that has been shown to be effective in lowering serum Phe concentrations and/or improving dietary Phe tolerance in a subset of patients with PKU or mild HPA who respond to treatment with BH_4_ (known as responders) and in the rare patients with a defect in BH_4_ synthesis [12]. Based on the results of the SPARK (Safety Paediatric efficAcy phaRmacokinetics with Kuvan®) study, the European Medicines Agency has recently extended the indication for sapropterin from the treatment of BH_4_-responsive PKU in adults and children aged ≥4 years and in all BH_4_-deficient adults and children [12, 13] to now include children with BH_4_-responsive PKU <4 years old, for whom the previous standard of care was a Phe-restricted diet.

The primary aim of the SPARK study was to evaluate the efficacy (increase in Phe tolerance, defined as the amount of Phe a patient may consume while maintaining blood Phe concentrations within the target range of 120–360 μmol/L); safety of 26 weeks of treatment with sapropterin dihydrochloride plus a Phe-restricted diet compared with a Phe-restricted diet alone in children <4 years of age with BH_4_-responsive PKU or mild HPA; to document the relationship between exposure and response; and to support the posology in this age-group. Although population pharmacokinetic (PopPK) data for sapropterin have been published for infants and young children in the USA and Canada [14], there are no PopPK data for sapropterin in this age range in the European Union (EU); therefore, a secondary aim of SPARK was to develop a PopPK model for sapropterin in this population. The other secondary endpoints were to document the concentrations of blood Phe during the study and extension periods, to document the change in dietary Phe tolerance, and to monitor blood pressure, growth parameters, and neuromotor developmental milestones.

## Methods

### Study design

The SPARK trial (NCT01376908) is a 26-week open-label, multicentre, randomized phase IIIb study to assess the efficacy, safety and PopPK of sapropterin in patients aged <4 years with BH_4_-responsive PKU or mild HPA. SPARK was conducted at 22 sites in nine countries: Austria (*n* = 2), Belgium (*n* = 2), Czech Republic (*n* = 1), Germany (*n* = 4), Italy (*n* = 5), Netherlands (*n* = 2), Slovakia (*n* = 3), Turkey (*n* = 1) and the United Kingdom (*n* = 2). The study was performed in accordance with the protocol and subsequent protocol amendments and with the ethical principles laid down in the Declaration of Helsinki, in accordance with the International Conference on Harmonisation (ICH), Note for Guidance on Good Clinical Practice (ICH Topic E6, 1996) and applicable regulatory requirements. The local ethics committee/institutional review board at each of the participating centres approved the protocol.

### Patients

Male or female patients aged <4 years at randomization were eligible for entry into the study if they had participated in the screening protocol <42 days before study day 1, had a confirmed diagnosis of mild HPA or PKU (a defined level of Phe tolerance consistent with a diagnosis of PKU, ≥2 previous blood Phe concentrations ≥400 μmol/L obtained on two separate occasions), were responsive to BH_4_ (a decrease of >30% in Phe concentrations following a 20 mg/kg BH_4_ challenge of at least 24 h), good adherence to dietary treatment and maintenance of blood Phe concentrations within the therapeutic target range (120–360 μmol/L) for 4 months prior to screening or at least the last four values of Phe (either from venous blood or dry blood spot) were to be assessed, out of which 75% had to be within the above therapeutic range. Patients were excluded if they had used sapropterin or any preparation of BH_4_ within the previous 30 days (unless for the purposes of the BH_4_ responsiveness test), had known hypersensitivity to sapropterin, its excipients, or to other approved or non-approved formulations of BH_4_, or had a previous diagnosis of BH_4_ deficiency.

Patients’ parent(s)/guardian(s) gave written informed consent for participation in the study before any trial-related procedures were performed. Parent(s) and/or guardian(s) had to be willing to comply with all study procedures, maintain strict adherence to the diet, and be willing and able to provide written, signed informed consent after the nature of the study had been explained and prior to any study procedures. Where required, separate informed consent was obtained from the patients’ parents or guardians to obtain samples for pharmacokinetic analysis.

### Randomization

**Fig. 1:**
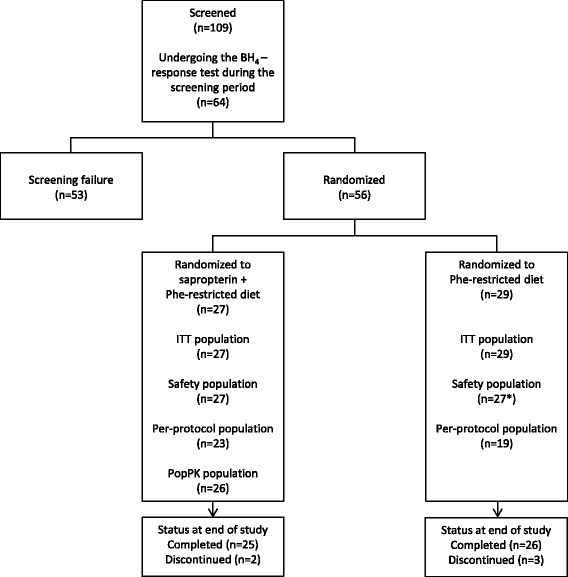
Patient disposition. *Two of the randomized patients withdrew consent after randomization. No safety assessments were performed during the study period

On study day one, patients were randomly assigned 1:1 to 10 mg/kg/day oral sapropterin dissolved in water to be taken with breakfast (after 4 weeks, sapropterin could be increased to 20 mg/kg/day if Phe tolerance had not increased by >20% vs. baseline) plus a Phe-restricted diet or only a Phe-restricted diet for 26 weeks. After study completion, patients were eligible to enrol in a 3-year extension period (to be reported separately), during which all patients received sapropterin plus a Phe-restricted diet (Fig. 1).

### Efficacy assessments

The primary outcome was an improvement in dietary Phe tolerance, defined as the daily amount of Phe (mg/kg/day) that could be ingested while sustaining mean blood Phe concentrations within a target range of 120–360 μmol/L by dietary Phe adjustments following an algorithm (Table 1). An additional supportive analysis was performed, in which dietary Phe tolerance was based on the Phe intake reported in a 3-day Phe diet diary used to monitor the adherence to the Phe-restricted diet. Analysis and adjustment of dietary intake were performed by the investigator and/or experienced dietician/nutritionist every 2 weeks during the study, according to the study algorithm.

**Table 1:** Algorithm for phenylalanine (Phe) intake adjustments according to mean Phe concentrations

Mean Phe concentration, μmol/L	Phe intake adjustment
*At Week 2*	
0–300	Increase by 5 mg/kg/day
301–359	No adjustment required
360–∞	No adjustment required, but monitor concentration at next visit
*Post-Week 2*	
0–180	Increase by 15 mg/kg/day
181–240	Increase by 10 mg/kg/day
241–300	Increase by 5 mg/kg/day
301–359	No change in dietary Phe intake
360–∞	Determine if the subject had one or more previous dietary Phe intake increases • If so, remove the dietary Phe in the order that it was previously increased, beginning with the amount of the last increase • If not, no adjustment required
360–1199	If this is first occasion at this concentration, monitor concentration at the next visit and if second occasion is at this concentration, provide dietary counseling
1200–∞	If this is first occasion at this concentration, provide dietary counseling and monitor concentration at the next visit and if second occasion is at this concentration, provide dietary counseling and terminate from the trial

Blood Phe concentrations were measured twice weekly via dried blood spot cards using a high-performance liquid chromatography/tandem mass spectrometry method for Phe detection. The results were verified every 3 months using venous blood plasma. Blood Phe samples could be obtained more frequently at the investigator’s discretion.

Secondary endpoints included neuromotor development and physical growth parameters (height or length, weight and maximal occipital-frontal head circumference). Neuropsychological development was assessed using the adaptive behaviour composite score with the Bayley III and the social-emotional composite score in the WPPSI-III, although these results are not reported in this manuscript.

### Pharmacokinetic analysis

The PopPK analysis population comprised all randomized subjects with ≥1 pharmacokinetic sample. PopPK parameters were apparent clearance (CL/F), apparent volume of distribution (V/F), absorption rate constant (K_a_), and endogenous BH_4_ (C0). These were used to compute the area under the curve (AUC_0–∞_), peak serum concentration (C_max_), time of C_max_ (T_max_), and half-life (t_1/2_). Plasma samples were collected for endogenous BH_4_ measurement at baseline and sparsely thereafter between weeks 5–12 after oral administration of sapropterin 10 mg/kg/day. In order to ensure that the sparse pharmacokinetic sampling provided sufficient information and that samples were taken at informative times, the sampling had been planned using D-optimization [15]. During this process, competing maturation functions were considered [16, 17].

PopPK modelling was conducted using NONMEM® (software version 7, level 2; Icon Development Solutions, Ellicott City, MD, USA) using standard model building and evaluation approaches. Covariates, including age, weight and sex, were evaluated using standard methodology to determine if these factors were predictive of BH_4_ pharmacokinetics. The final model was subsequently used to derive metrics of exposure and to determine the exposure relative to adult PKU patients.

### Laboratory assessments

All standard blood chemistry, hematologic and urine analysis, as well as specialized testing for Phe and tyrosine concentrations, were performed at a central laboratory.

### Safety analysis

The safety population consisted of all subjects who had some safety assessment data available. Safety was assessed at the clinic on a monthly basis during the 26-week study period or until 4 weeks post-treatment, by recording, reporting, and analysis of baseline medical conditions and adverse events (AEs) and physical examination findings (including vital signs). Standard blood chemistry, hematologic, and urine analyses were performed every 3 months during the study period for safety analysis.

### Genotype analysis

*PAH* genotype data were collected at screening for enrolled patients, after a separate informed consent was obtained from the patients’ parents or guardians. Genotype testing was performed by a central laboratory.

### Statistical analyses

The primary efficacy analysis population was the intention-to-treat (ITT) population comprising all randomized patients. The per-protocol (PP) population included all ITT patients who completed the study with no prohibited concomitant medication and without major protocol deviation. A missing pre-study Phe tolerance, lack of adherence to Phe-restricted diet over the past 3 months, lack of adherence to sapropterin, and a sapropterin-dose adjustment not conducted as per protocol were considered to be major protocol deviations leading to exclusion from the PP population. The safety population comprised all patients with safety assessment data available (≥1 visit for vital signs, AEs or laboratory results) and who had received ≥1 dose of sapropterin or were randomly assigned to Phe-restricted diet alone.

The sample size was planned to be 23 patients per group, to ensure a power of 80% to demonstrate a treatment group difference, assuming a dietary Phe tolerance of 20 mg/kg/day under dietary therapy alone, a difference of 75% with the sapropterin plus diet group, and a common standard deviation of 17.5 mg/kg/day. To compensate for possible dropouts, a total of 50 subjects were to be randomized.

The dietary Phe tolerance was analyzed using the repeated measures analysis of covariance (ANCOVA) on the observed records for the ITT population, with baseline Phe tolerance, treatment group, age group, visit, baseline blood Phe concentration and treatment by visit interaction as fixed effects. Secondary endpoints were described using summary statistics.

Non-linear mixed-effect modelling (NONMEM® software version 7, level 2) was applied to estimate the pharmacokinetic parameters and their variability. The final model was evaluated using a number of methods, which included bootstrapping and visual predictive checks, as conducted previously in children aged 0–6 years [18].

In order to evaluate the differences in exposure expected from the original model and the current model, simulated concentration–time profiles for the reference subject were generated.

## Results

### Patient disposition and demographics

In total, 109 patients were screened (Table 2 and Fig. 1), of whom 53 were screening failures (49 patients did not meet eligibility criteria and four patients for other reasons). Fifty-six patients were randomized (27 patients to the sapropterin plus Phe-restricted diet group and 29 patients to the diet-only group). Fifty-two patients were included in the PopPK population. Patients were stratified according to age: 15 patients were <12 months, 18 patients were 12 to <24 months, and 23 patients were 24 to <48 months. A numerically higher proportion of patients in the sapropterin plus Phe-restricted diet group successfully followed the protocol compared with patients in the Phe-restricted diet only group (85% [23 of 27 patients] vs. 65% [19 of 29 patients]). The mean (±standard deviation [SD]) age at diagnosis was 30 (±75.3) days. Almost half (46.4%) were diagnosed with mild HPA, 32.1% were diagnosed with mild PKU, and 21.4% were diagnosed with classical PKU.

**Table 2:** Demographic and baseline characteristics (ITT population)

Characteristic	Sapropterin + Phe- restricted diet (*n* = 27)	Phe-restricted diet only (*n* = 29)
Age, months		
mean±SD	21.1±12.3	21.2±12.0
min; max	2; 47	2; 44
Age group, n (%)		
<12 months	7 (25.9)	8 (27.6)
12 − <24 months	9 (33.3)	9 (31.0)
24 − <48 months	11 (40.7)	12 (41.4)
Males, n (%)	16 (59.3)	14 (48.3)
Height, cm		
mean±SD	82.0±11.3	82.3±11.6
min; max	59; 108	57; 105
Weight, kg		
mean±SD	11.3±3.1	11.3±2.8
min; max	5; 20	6; 16
BMI, kg/m^2^		
mean±SD	16.5±1.0	16.5±1.4
min; max	14; 18	14; 20
Age at PKU diagnosis, days		
mean±SD	27.2±79.8	32.6±72.2
min; max	1; 425	4; 382
Blood Phe concentration at diagnosis, μmol/L		
mean±SD	780.3±480.7	879.9 ± 596.5
min; max	191; 2062	221; 2600
PKU severity^a^ n (%)		
Classical PKU	5 (18.5)	7 (24.1)
Mild PKU	10 (37.0)	8 (27.6)
Mild HPA	12 (44.4)	14 (48.3)

*BMI* body mass index, *HPA* hyperphenylalaninemia, *ITT* intention-to-treat, *Phe* phenylalanine, *PKU* phenylketonuria, *SD* standard deviation

^a^Disease severity according to blood Phe concentrations: Classical PKU, >1200 μmol/L; Mild PKU, 600–1200 μmol/L; Mild HPA, 120–600 μmol/L^2, 5, 6^

The overall mean adherence to sapropterin (defined as the proportion between the actual dose administered and the prescribed dose) over the study was 100% (range 82 to 107%). Most patients (*n* = 25, 92.6%) continued on 10 mg/kg/day after 4 weeks of treatment, with only two patients switching to 20 mg/kg/day. The overall mean (±SD) adherence to diet, as assessed by a 3-day food diary, was 94.6±9.4% (range 69 to 111%) in the sapropterin-treated group and 92.1±23.8% (range 65 to 183%) in the diet-only treated group.

### Dietary Phe tolerance after 26 weeks

**Fig. 2:**
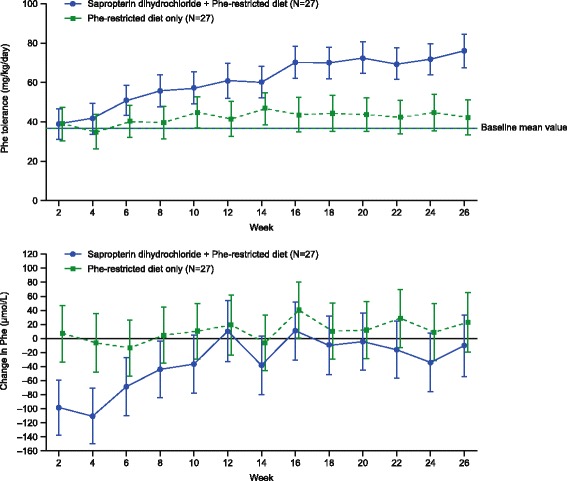
**a** Adjusted mean dietary Phe tolerance (mg/kg/day) **a** and mean Phe change from baseline (μmol/L) **b** Error bars represent 95% confidence intervals. Phe, phenylalanine. CI, confidence interval; Phe, phenylalanine

At week 26, the adjusted mean dietary Phe tolerance was higher in the sapropterin plus Phe-restricted diet group compared with the diet-only group. The tolerance based on prescribed Phe was 80.6 mg/kg/day vs. 50.1 mg/kg/day (adjusted between-group difference 30.5 mg/kg/day [95% confidence interval (CI) 18.7, 42.3], *p* < 0.001). The tolerance based on reported dietary Phe tolerance from the intake diary was 75.7 mg/kg/day [95% CI 67.2, 84.11] vs. 42.0 mg/kg/day [95% CI 33.1, 50.8] (adjusted between-group difference 33.7 [95% CI 21.4, 45.9], *p* < 0.001; Fig. 2a). A similar difference was reported in the per-protocol population (adjusted between-group difference 36.4 [95% CI 25.4, 47.4], *p* < 0.001). In addition, consistent results were seen in the ITT population following supportive analysis on diary-recorded Phe intake.

### Blood Phe concentrations

Phe concentrations from dried blood spots were lower than those from venous blood spots but this was consistent with differences reported in the literature [19–21]. In the Phe-restricted diet group, the adjusted mean blood Phe concentrations in the ITT population were stable over time, with a mean (±SD) increase of 23.1 (±21.9) μmol/L at week 26 (Fig. 2b). In the sapropterin plus Phe-restricted diet group, the mean (±SD) blood concentrations decreased by 110.7 (±20.1) μmol/L at week 4 and gradually returned to concentrations similar to those seen in the Phe-restricted diet group, reflecting the increase in Phe intake and Phe tolerance. At week 26, the adjusted mean (±SD) blood Phe concentrations were similar: 300.1 (±115.2) μmol/L in the sapropterin plus Phe-restricted diet group and 343.3 (±118.4) μmol/L in the diet-only group (adjusted between-group difference 33.2 μmol/L [95% CI −94.8, 28.4], *p* = 0.290). It is important to note that patients were expected to maintain blood Phe concentrations within this range; therefore, differences in blood Phe concentrations were not anticipated.

The observed proportion of patients with blood Phe concentrations maintained in the range 120–360 μmol/L throughout the whole study was greater in the sapropterin plus Phe-restricted diet group (*n* = 9/27, 33.3%) than in the diet-only group (*n* = 3/29, 10.3%). 21 of 27 (77.8%) sapropterin-treated patients and 15 of 27 (55.6%) patients on only the Phe-restricted diet had ≥1 blood Phe concentration at or below the 120 μmol/L threshold established by the British PKU Registry [22]. However, very few instances of Phe concentration below the normal range thresholds of 40 and 26 μmol/L were observed during the study.

### Change from baseline in dietary Phe tolerance

The mean change in dietary Phe tolerance between baseline and the last Phe tolerance observation was assessed within each treatment group. The mean (±SD) change from baseline to week 26 in patients receiving sapropterin plus Phe-restricted diet was 36.9 (±27.3) mg/kg/day (*p* < 0.001). The mean change from baseline in patients only on the Phe-restricted diet was 13.1 (±19.6) mg/kg/day (*p* = 0.002).

### Pharmacokinetic analysis

The pharmacokinetic data are best described by a one-component model with first-order input following a time lag and first-order elimination, with an endogenous baseline BH_4_ concentration component. The model included terms describing between-subject variability on apparent clearance (CL/F) and apparent volume of distribution (V/F) as well as their correlation (Table 3). The final model parameter estimate for CL/F was 2780 L/h, 3870 L for V/F, and 0.234 h^−1^ for K_a_.

**Table 3:** Parameter estimates for final model

	Population mean	SE%
CL/F (L/h)	2780	2.0
V/F (L)	3870	5.9
K_a_ (1/h)	0.234	6.6
LAG (h)	0.342	2.8
C0 (μg/L)	12.6	7.8
Coefficient describing effect of weight on CL/F	0.839	1.8
Coefficient describing effect of weight on V/F	0.573	3.3
Residual error (%CV)	65.30	8.5
IIV_CL (%CV)	22.98	0.2
IIV_V2 (%CV)	32.56	0.2
Corr (CL,V)	0.134	NE

*SE* standard error, *CL/F* apparent clearance, *V/F* apparent volume of distribution, *LAG*, lag time, *K*_*a*_ absorption rate constant, *C0* endogenous BH_4_ concentrations, *CV* coefficient of variation, *IIV* between-subject variability, *NE* not estimated

From the model, an elimination half-life of approximately 1 h can be computed, with an absorption half-life (ln2/K_a_) of approximately 3 h, suggesting flip-flop kinetics where the absorption becomes the rate-limiting step of drug disposition.

**Fig. 3:**
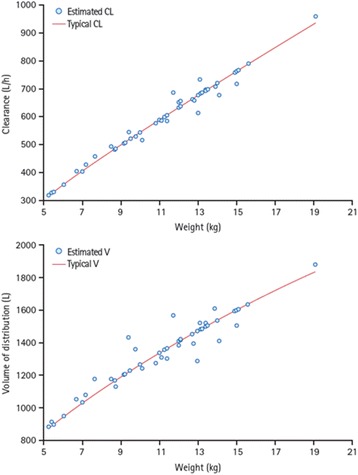
Relationship between weight and clearance **a** and weight and volume of distribution **b**

Body weight was the only covariate that affected the CL/F and V/F of sapropterin: these variables increased in a nonlinear manner with increasing weight, although individual predictions still varied around the typical individual predictions (Fig. 3). At the lowest extreme of weight, a 5 kg patient had a CL/F value 11% of that of a 70 kg reference adult, and a V/F value 22% of that of the reference adult (Table 4).

**Table 4:** Effect of weight on clearance and volume of distribution

Weight (kg)	CL/F (L/h)	% of reference	V/F (L)	% of reference
5	305	10.9	853	22.0
15	766	27.5	1601	41.4
25	1176	42.2	2145	55.4
70^a^	2789	100.0	3870	100.0

*CL/F* apparent clearance, *V/F* apparent volume of distribution

^a^Reference weight (adult male patient)

**Fig. 4:**
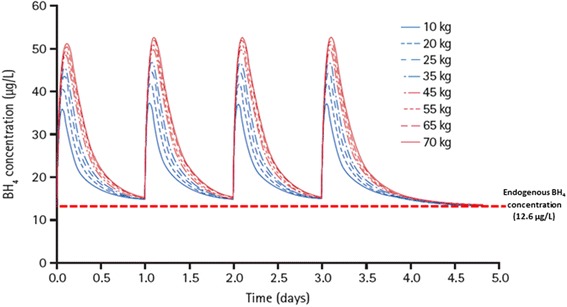
Simulated concentration–time curves for patients with various weights following sapropterin (10 mg/kg/day)

Even after inclusion of weight into the pharmacokinetic model, significant between-subject variability in CL/F and V/F remained, supporting an adaptive approach to individual treatment. Simulated concentration–time curves following sapropterin 10 mg/kg show that sapropterin concentrations remain above the model-estimated endogenous BH_4_ concentrations (12.6 μg/L; Table 3) for the dose interval for patients with different weights (Fig. 4).

Overall, the exposure across all age groups is comparable, although the number of patients in all age groups is small. The exposure in pediatric patients was lower than the expected exposure in adults, based on the simulated concentration–time profiles following the 10 mg/kg/day dose across a range of body weights. This analysis shows that the concentrations remain above the endogenous concentration, which is set at a concentration below that for a person not diagnosed with PKU, for a daily dose interval and support the current approach to treatment as conservative (Fig. 4).

### Safety

The safety population comprised 54 patients; two of the randomized patients withdrew consent after randomization and, therefore, were excluded from the safety population (Fig. 1). All patients in the safety population reported at least one AE (Table 5); in the sapropterin plus Phe-restricted diet group, eight out of 27 patients (29.6%) reported at least one treatment-emergent AE (TEAE) classified as related to sapropterin. The proportion of patients reporting TEAEs was the same in the two groups, and no patients withdrew owing to AEs. None of the TEAEs were graded as severe. All patients had at least one TEAE that was judged to be mild in severity. Seven (25.9%) patients in the sapropterin plus Phe-restricted diet group had nine TEAEs graded as moderate in severity, and eight (29.6%) patients in the Phe-restricted diet group reported 18 TEAEs graded as moderate in severity.

**Table 5:** Summary of safety data showing the proportion of patients reporting adverse events (AEs) (Safety population)

	Sapropterin + Phe-restricted diet (*n* = 27)		Phe-restricted diet alone (*n* = 27)	
	Patients, n (%)	Events, n	Patients, n (%)	Events, n
Treatment-emergent AEs	27 (100)	282	27 (100)	278
AEs related to sapropterin	8 (29.6)	31	NA	NA
Infections and infestations related to sapropterin	3 (11.1)	3	NA	NA
Gastrointestinal disorders related to sapropterin	3 (11.1)	8	NA	NA
Amino acid concentrations decrease related to sapropterin	6 (22.2)	20	NA	NA
SAEs	3 (11.1)	5	1 (3.7)	2
Gastroenteritis	1 (3.7)	1	0 (0.0)	0
Rash	1 (3.7)	1	0 (0.0)	0
Overdose^a^	1 (3.7)	2	0 (0.0)	0
Stomatitis	1 (3.7)	1	0 (0.0)	0
Bronchiolitis	0 (0.0)	0	1 (3.7)	1
Bronchopneumonia	0 (0.0)	0	1 (3.7)	1
AEs leading to discontinuation	0 (0.0)	0	0 (0.0)	0

*NA* not appropriate, *SAE* serious AE

^a^On the day of first administration of study treatment, the subject had a sapropterin overdose (severity: mild; 80 mg/day instead of 75 mg/day by mistake). At 26 days after the first administration of study treatment, the subject had another sapropterin overdose (severity: mild; 80 mg/day instead of 75 mg/day by mistake). Both events were reported in accordance with the protocol and were therefore categorized as medically important. The subject recovered without sequelae from both events. The administration of sapropterin plus Phe-restricted diet alone was continued without change after the first overdose and the dose was reduced after the second overdose

AE defined as any untoward medical occurrence in a subject or clinical investigation subject administered a pharmaceutical product, which did not necessarily have a causal relationship with trial treatment; SAE was any untoward medical occurrence that at any dose: resulted in death; was life-threatening; might have caused death if it had been more severe; required inpatient hospitalization or prolongation of existing hospitalization; resulted in persistent or significant disability/incapacity; was a congenital anomaly/birth defect; or was otherwise considered as medically important

The most common TEAEs in the sapropterin plus Phe-restricted diet group and in the Phe-restricted diet group were: pyrexia (63.0 and 66.7%), cough (48.1 and 48.1%) and nasopharyngitis (48.1 and 40.7%), respectively. The most common TEAEs classified as related to sapropterin were amino acid concentration decrease (six patients [22.2%]), rhinitis, and vomiting (two patients each [7.4%]), and one patient (3.7%) each for pharyngitis, diarrhea, abdominal pain, mouth ulceration and increased amino acid concentration.

Although the proportion of patients who reported a serious AE (SAE) was higher in the sapropterin plus Phe-restricted diet group compared with the Phe-restricted diet (11.1 vs. 3.7%), all SAEs were assessed as unrelated to sapropterin treatment (Table 5).

### Genotype data

Of 109 patients who were screened, 73 agreed to participate in the pharmacogenetics sub-study. Of the 73 patients who agreed, 36 were screening failures, leaving genotype data for 37 responders (Additional file 1: Table S1).

### Neuromotor development and growth parameters

Most patients in both treatment groups had normal neuromotor development, including fine motor, gross motor, language, and personal and social function, and there were no statistically significant differences between treatment groups in any of the neuromotor developmental milestones at baseline, 12 and 26 weeks (Additional file 1: Figure S1).

Patients in both treatment groups had stable growth parameters, including body mass index SD score (SDS), height SDS, maximum occipital-frontal head circumference SDS and weight SDS. There were no statistically significant differences between the treatment groups for any of the growth parameters.

## Discussion

In PKU, blood Phe concentrations need to be controlled from birth to prevent neurological sequelae, such as cognitive impairment and mild-to-severe intellectual disability, linked to PKU [5, 7]. Until July 2015 there was no licensed pharmacological treatment available in the EU for children with PKU aged <4 years, and the standard of care was a Phe-restricted diet. The results of the SPARK study, which was the first clinical trial of sapropterin in patients 0–4-years-old with BH_4_-responsive PKU or mild HPA in Europe, showed that daily dosing with 10 or 20 mg/kg/day sapropterin in combination with a Phe-restricted diet led to statistically and clinically significant improved dietary Phe tolerance at week 26 compared with a Phe-restricted diet alone, while maintaining mean blood Phe concentrations within the protocol-specified range. These results were consistent with those seen in children aged 4–12 years treated with 20 mg/kg/day sapropterin, in whom the mean amount of Phe supplement tolerated had increased at 10 weeks of treatment [23]. The results were also consistent with those reported in a study from the USA and Canada in children aged 0–6 years old, in whom 20 mg/kg/day sapropterin treatment lowered blood Phe concentrations, enabling, in some cases, an increase in dietary Phe intake [24].

The benefits of initiating sapropterin therapy in patients younger than 4 years have been highlighted by a post-marketing study conducted in Japan between 1995 and 2001, which reported that all patients who started treatment with sapropterin before the age of 4 years maintained serum Phe concentrations within the recommended range for the duration of the study [25]. Previous reports have shown that neurocognitive function was preserved and no neurodevelopmental penalty was reported in patients who started sapropterin therapy between 0 and 6 years of age [24], and that treatment with BH_4_ may enable relaxation of the dietary regimen, leading to improved quality of life [26]. Patients with mild HPA, who comprised almost a half of the population in this study, retain substantial enzyme activity and will, therefore, likely respond to sapropterin treatment. However, the indication for treatment of mild HPA differs between countries due to weak evidence. US guidelines recommend treatment at a Phe concentration above 360 μmol/L [10], while other countries start treatment at Phe concentrations above 600 μmol/L [27].

In this study, the addition of sapropterin to a Phe-restricted diet in patients <4 years old with BH_4_-responsive PAH deficiency significantly improved Phe tolerance compared with a Phe-restricted diet alone. In the sapropterin-treated group, blood Phe concentrations initially fell at the beginning of treatment (4 weeks), but they slowly increased over the course of the study to reach concentrations similar to those at baseline by week 12 (Fig. 2), while increasing dietary Phe-intake. The observed increase in Phe tolerance reported in patients on the Phe-restricted diet compared with the tolerance at baseline may be explained by the fact that the patients in this group were not at their maximum Phe tolerance in daily practice before starting the study. This observation confirms the expectation that under the tight control of study conditions using a strict algorithm of Phe escalation, dietary Phe tolerance may be further optimized [28]. Because of the potential for Phe concentrations to drop below either the normal or the desired therapeutic concentrations owing to the action of sapropterin, careful monitoring and adjustment of therapeutic dose and dietary Phe concentrations was necessary.

The pharmacokinetics of BH_4_ can be well described by a one-compartment model that respects the principle of parsimony and provides accurate estimates that describe BH_4_ profiles virtually identical to those from a two-compartment model evaluated in a previous study [18]. The terminal and absorption half-lives are suggestive of flip-flop pharmacokinetic behavior, in which absorption is the rate-limiting step of drug disposition. Sapropterin exposure was similar across all age groups studied here. With this in mind, a once-daily dosing regimen is justified. Weight was the only covariate that had an effect on the clearance and volume distribution of sapropterin, meaning that dose adjustments based on weight are appropriate [14].

The secondary endpoints of growth and neuromotor development were considered to be normal in the patient population throughout the study and no difference between groups was observed, suggesting no treatment effect on these growth and development parameters. However, the time scale in the study was too short to expect clinically meaningful changes in neuromotor development.

The safety profile for sapropterin was acceptable and similar to that reported in studies of patients >4 years old [23] and in those <4 years old [25], with no deaths, severe TEAEs or withdrawals reported. Although four patients had SAEs, none of these was deemed to be related to treatment. The number of TEAEs was similar between the two groups and was commonly associated with normal childhood illness.

## Conclusion

In conclusion, the addition of sapropterin to a Phe-restricted diet in patients aged <4 years old with BH_4_-responsive PKU, mild PKU or mild HPA was well tolerated and led to a significant improvement in Phe tolerance compared with only a Phe-restricted diet. The pharmacokinetics of sapropterin in patients aged <4 years are adequately described by a one-compartment model, and favor once-a-day dosing with dose adjustment for weight. These data led to the approval of sapropterin for individuals with BH_4_-responsive PKU or mild HPA aged <4 years, and will thus change treatment management for this subset of patients in the first week of life.

## Additional file

Additional file 1: Figure S1. Summary of neuromotor developmental milestones – ITT population. Data show (a) the proportion of patients with normal development in the area of assessment at baseline, (b) Week 12, and (c) Week 26 treated with sapropterin plus the Phe-restricted diet, or Phe-restricted diet alone. *P*-values show comparison of results between treatment groups at Week 26 using the chi-squared test. **Table S1**. *PAH* genotypes of sapropterin responders (*n*=37). (DOCX 180 kb)

## Abbreviations

AE: Adverse event
ANCOVA: Analysis of covariance
AUC: Area under the curve
C0: Endogenous BH_4_
C_max_: Peak serum concentration
CI: Confidence interval
CL/F: Apparent clearance
EU: European Union
HPA: Hyperphenylalaninemia
ICH: International Conference on Harmonisation
ITT: Intention-to-treat
K_a_: Adsorption rate constant
PAH: Phenylalanine hydroxylase
Phe: Phenylalanine
PKU: Phenylketonuria
PopPK: Population pharmacokinetics
PP: Per-protocol
SAE: Serious AE
SD: Standard deviation
SDS: SD score
SPARK: Safety paediatric efficacy pharmacokinetics with Kuvan®
T_1/2_: Half-life
T_max_: Time of C_max_
TEAE: Treatment-emergent AE
V/F: Apparent volume of distribution
