# Supplementary material for: Efficacy, safety and population pharmacokinetics of sapropterin in PKU patients <4 years: results from the SPARK open-label, multicentre, randomized phase IIIb trial
Source: Orphanet J Rare Dis. 2017 Mar 9;12:47. doi: 10.1186/s13023-017-0600-x (PMC5343543; doi:10.1186/s13023-017-0600-x)
Supplement: Additional file 1: Figure S1. — Summary of neuromotor developmental milestones – ITT population. Data show (a) the proportion of patients with normal development in the area of assessment at baseline, (b) Week 12, and (c) Week 26 treated with sapropterin plus the Phe-restricted diet, or Phe-restricted diet alone. P-values show comparison of results between treatment groups at Week 26 using the chi-squared test. Table S1. PAH genotypes of sapropterin responders (n=37). (DOCX 180 kb) [file 13023_2017_600_MOESM1_ESM.docx]

**Additional file 1**

**Figure S1.** Summary of neuromotor developmental milestones – ITT population. Data show (a) the proportion of patients with normal development in the area of assessment at baseline, (b) Week 12 , and (c) Week 26 treated with sapropterin plus the Phe-restricted diet, or Phe-restricted diet alone. P-values show comparison of results between treatment groups at Week 26 using the chi-squared test

**a)
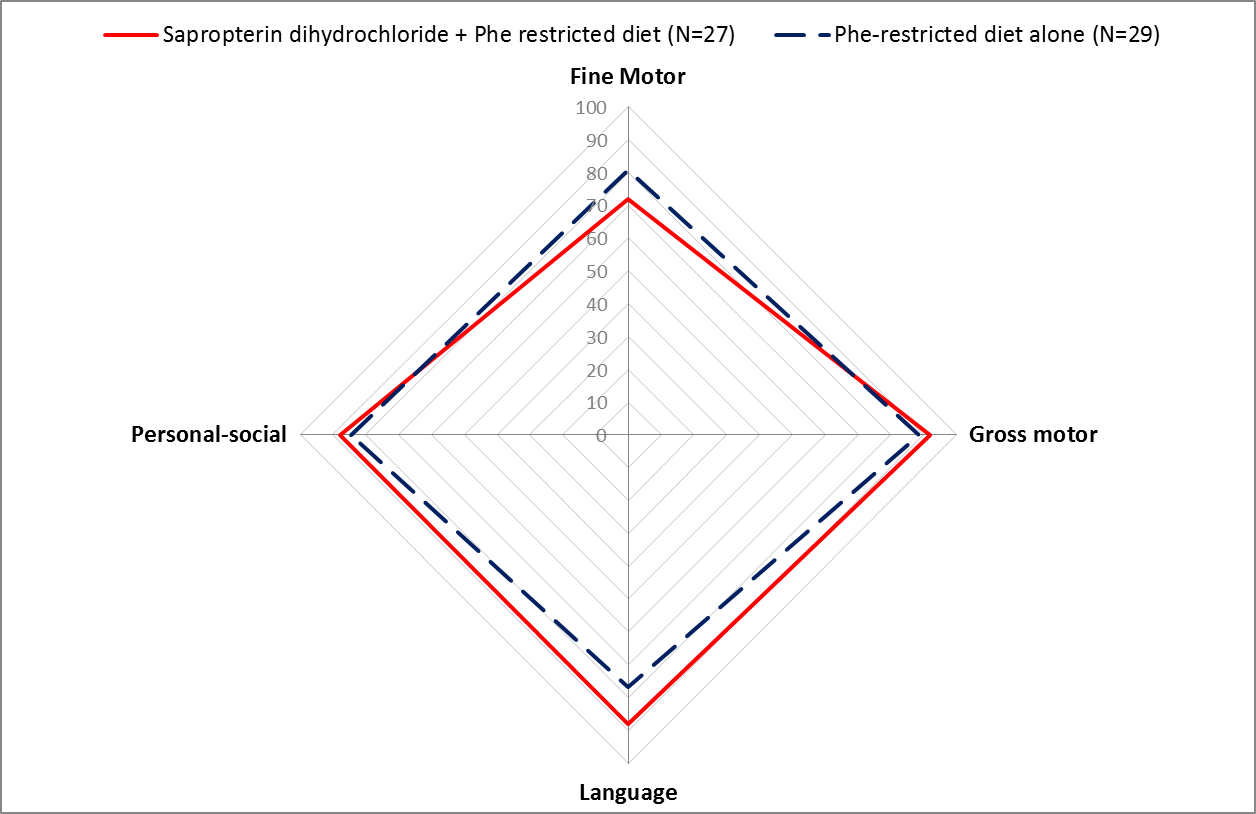
**

**b)
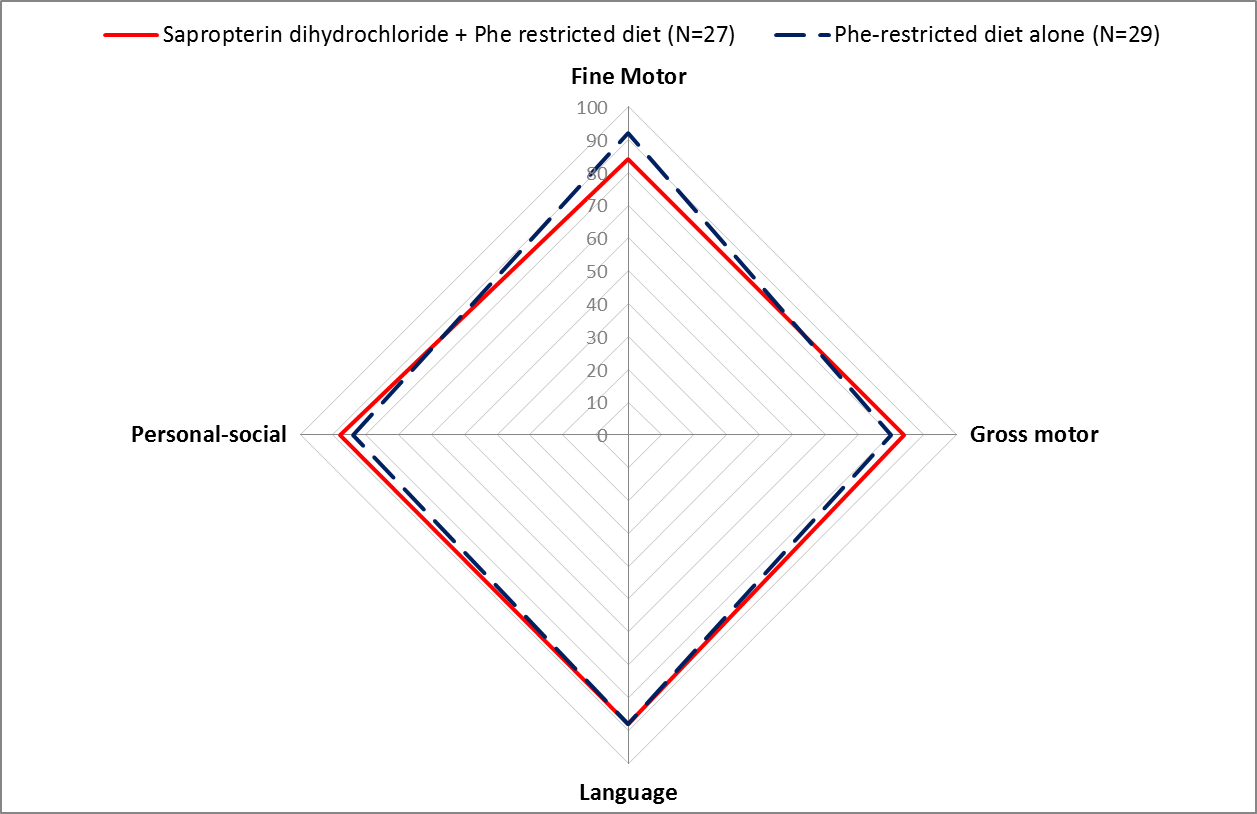
**

**c)
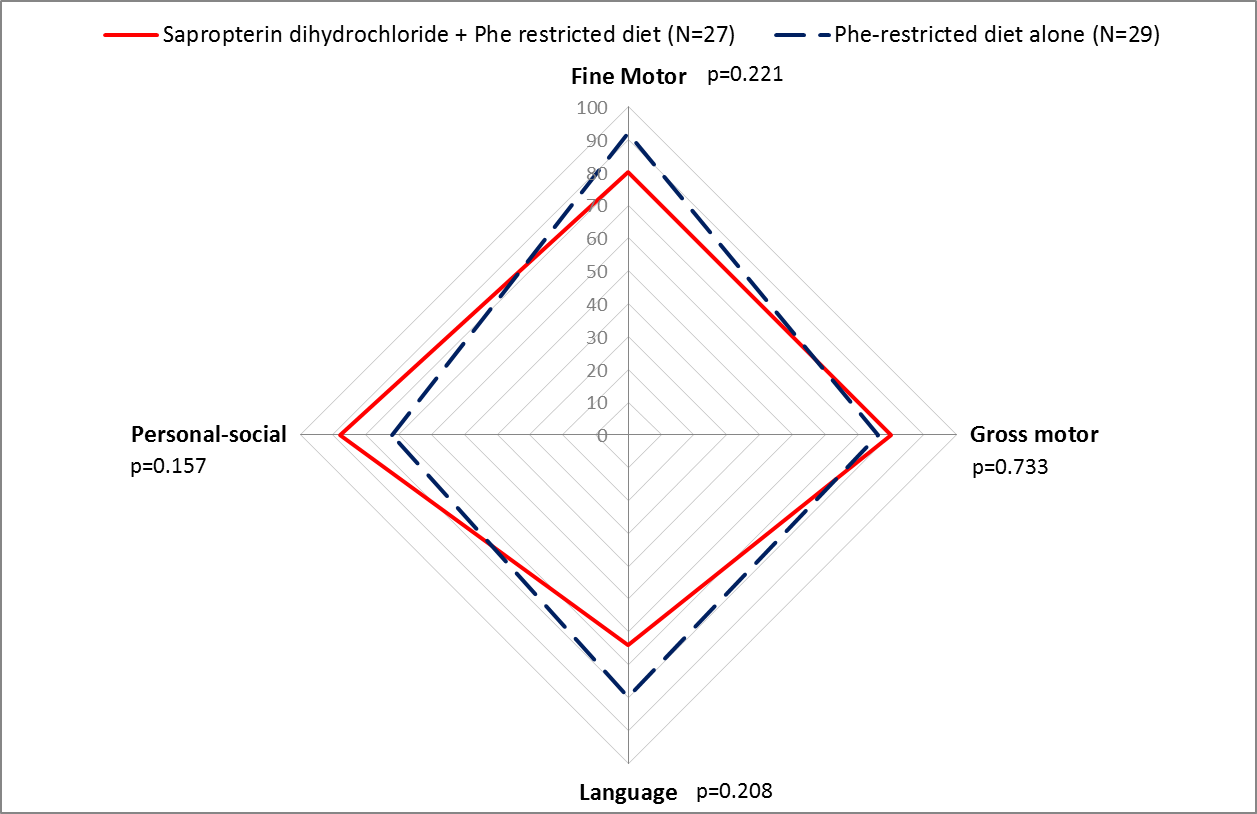
**

**Table S1.** Algorithm for phenylalanine (Phe) intake adjustments according to mean Phe levels

| **Mean Phe level, μmol/L** | **Phe intake adjustment** |
| --- | --- |
| ***At Week 2*** | |
| 0–300 | Increase by 5 mg/kg/day |
| 301–359 | No adjustment required |
| 360–∞ | No adjustment required, but monitor level at next visit |
| ***Post-Week 2*** | |
| 0–180 | Increase by 15 mg/kg/day |
| 181–240 | Increase by 10 mg/kg/day |
| 241–300 | Increase by 5 mg/kg/day |
| 301–359 | No change in dietary Phe intake |
| 360–∞ | Determine if the subject had one or more previous dietary Phe intake increases   - If so, remove the dietary Phe in the order that it was previously increased, beginning with the amount of the last increase - If not, no adjustment required |
| 360–1199 | If this is first occasion at this level, monitor level at the next visit and if second occasion is at this level, provide dietary counseling |
| 1200–∞ | If this is first occasion at this level, provide dietary counseling and monitor level at the next visit and if second occasion is at this level, provide dietary counseling and terminate from the trial |

**Table S2.** *PAH* genotypes of sapropterin responders (n=37)

| **Allele 1** | **Allele 2** | **Allele 3** | **Nucleotide changes** | **n** |
| --- | --- | --- | --- | --- |
| p.Y414C | c.1066-11G>A |  | c.[1066-11G>A(;)1241A>G] | 1 |
|  | c.1315+1G>A |  | c.[1241A>G(;)1315+1G>A] | 2 |
|  | p.L48S |  | c.[143T>C(;)1241A>G] | 1 |
|  | p.R408W |  | c.[1222C>T(;)1241A>G] | 2 |
|  | p.R158W |  | c.[472C>T(;)1241A>G] | 1 |
|  | c.842+1G>A |  | c.[842+1G>A(;)1241A>G] | 1 |
|  | p.I65T |  | c.[194T>C(;)1241A>G] | 1 |
|  | p.R158Q |  | c.[473G>A(;)1241A>G] | 1 |
| c.1066-11G>A | p.L48S |  | c.[143T>C(;)1066-11G>A] | 2 |
|  | p.R408Q |  | c.[1066-11G>A(;)[1223G>A] | 1 |
|  | p.E390G |  | c.[1066-11G>A(;)1169A>G] | 1 |
|  | p.Q267* |  | c.[799C>T(;)1066-11G>A] | 1 |
|  | p.F39deI |  | c.[116_118del(;)1066-11G>A] | 1 |
|  | p.A300S |  | c.[898G>T(;)1066-11G>A] | 1 |
|  | c.1066-7C>A |  | c.[1066-7C>A(;)1066-11G>A] | 1 |
| c.1315+1G>A | p.E178G |  | c.[533A>G(;)1315+1G>A] | 1 |
|  | p.N167I |  | c.[500A>T(;)1315+1G>A] | 1 |
|  | p.R68S |  | c.[204A>T(;)1315+1G>A] | 1 |
|  | c.1315+1G>A |  | c.[1315+1G>A];[=] | 1 |
| p.L48S | Homozygote |  | c.[143T>C];[143T>C] | 1 |
|  | p.V388M |  | c.[143T>C(;)1162G>A] | 1 |
| p.R408W | Homozygote |  | c.[1222C>T]; [1222C>T] | 1 |
|  | p.K320N |  | c.[960G>C(;)1222C>T] | 1 |
|  | p.D394H |  | c.[1180G>C(;)1222C>T] | 1 |
| p.R261Q | Homozygote |  | c.[782G>A];[782G>A] | 1 |
|  | p.H107P |  | c.[320A>C(;)782G>A] | 1 |
|  | p.I65T |  | c.[194T>C(;)782G>A] | 1 |
| p.R408Q | Homozygote |  | c.[1223G>A];[1223G>A] | 1 |
| p.E178G | p.P211T |  | c.[533A>G(;)631C>A(;)776C>T] | 1 |
| p.P69R | p.R297H | G352Vfs*48 | c.[206C>G(;)890G>A(;)1055delG] | 1 |
| p.D222G | c.169-?_352+?del |  | c.[665A>G(;)169-?_352+?del] | 1 |
| p.R252W | p.A403V |  | c.[754C>T(;)1208C>T] | 1 |
| c.442-5C>G | p.P281L |  | c.[442-5C>G(;)842C>T] | 1 |
| p.G272* | p.S349P |  | c.[814G>T(;)1045T>C] | 1 |
| n=number of genotypes | | | | |
|  | Most frequent mutations in at least 5 patients | | | |
|  | Mutations in 2–4 patients | | | |
|  | Genotypes in at least 2 patients | | | |
